# Prolonged excretion of type-2 poliovirus from a primary immune deficient patient during the transition to a type-2 poliovirus-free world, Israel, 2016

**DOI:** 10.2807/1560-7917.ES.2016.21.47.30408

**Published:** 2016-11-24

**Authors:** Merav Weil, Lester M Shulman, Sophia Heiman, Tali Stauber, Jacqueline Alfandari, Leah Weiss, Ilana Silberstein, Viki Indenbaum, Ella Mendelson, Danit Sofer

**Affiliations:** 1 Central Virology Laboratory, Public Health Services, Israel Ministry of Heath, at Sheba Medical Center, Tel Hashomer, Israel; 2 These authors contributed equally to this work; 3 Department of Epidemiology and Preventive Medicine, School of Public Health, Sackler Faculty of Medicine, Tel Aviv University, Tel Aviv, Israel; 4 Pediatric Department A and Immunology Service, Jeffrey Modell Foundation Center, Edmond and Lily Safra Children's Hospital, Sheba Medical Center, Tel Hashomer, Israel

**Keywords:** clinic, epidemiology, healthcare-associated infections, infection control, poliomyelitis, poliovirus, public health policy, surveillance, viral infections

## Abstract

Wild poliovirus type-2 has been eradicated, use of live type-2 vaccine has been terminated globally, and all type-2 polioviruses are under strict laboratory containment protocols. Re-emergence may arise from prolonged asymptomatic excretion of poliovirus by hospitalised primary immune deficient (PID) patients, as described here, through repeated exposure of close contacts to high titres of infected material. At this transition time, PID patients should be screened and hospital containment protocols updated in parallel with laboratory containment.

Wild poliovirus type 2 (WPV2) was formally declared eradicated in September 2015 [1]. In April 2016, there was a globally coordinated replacement of trivalent oral poliovirus vaccine (tOPV) with bivalent OPV (bOPV) which lacks the type-2 poliovirus vaccine strain [2]. In July 2016, the Global Action Plan III (GAP III) [3], a protocol specifically designed to minimise the risk for re-emergence of type-2 poliovirus (PV2) from laboratory sources, restricted work and storage of PV2, and any materials that potentially contained this virus to annually certified ‘essential’ poliovirus laboratories that meet strict containment and biosafety standards. However, PV2 may re-emerge during this time from an alternative source for which there is no corresponding GAP III protocol, namely, prolonged infections with OPV type 2 (OPV2) in primary immune deficiency patients (PIDs) especially in closed hospital settings.

We present identification by chance of a prolonged PV2 infection in a primary immune deficient child in Israel during the global transition to a PV2-free world. This report serves to raise public health awareness of the implications for re-emergence of PV2.

## Primary immune deficient case with a prolonged poliovirus infection

A young non-Israeli child received a routine dose of tOPV at 2 months of age in the country of residence. Because of failing to thrive and frequent infections, the child was hospitalised at 5 months of age in Israel with a suspected diagnosis of severe immune deficiency. Fluorescence-activated cell sorting (FACS) analysis confirmed T-B-NK + immune phenotype, and genetic evaluation revealed a homozygote DNA cross-link repair 1C (*DCLRE1C*) gene mutation, leading to a final diagnosis of severe combined immune deficiency (SCID). The child was placed in reverse isolation, was started on antibiotic prophylaxis and received intravenous immunoglobulin (IVIG) once a month. At 8 months of age, the child received a haploidentical haematopoietic stem cell transplantation (HSCT). Microsatellite analysis 8 months post-bone marrow transplant (BMT) to evaluate engraftment suggested transplant failure. Currently, the child is awaiting a second transplantation.

After confirmation of SCID and before the HSCT, a stool sample was sent for viral diagnosis of transient diarrhoea to the National Poliovirus and Enterovirus Laboratory. It was positive for enterovirus by real-time reverse transcription-polymerase chain reaction (RT-PCR) and the virus had a cytopathic effect (CPE) on L20B cells suggesting poliovirus.

Viral protein 1 (VP1) sequence typing [4,5] identified the enterovirus as a type-2 vaccine derived poliovirus (VDPV2) with nine nucleotide (nt) and five amino acid substitutions. Single nt misincorporations accumulate at a rate of ca 1% per year as polioviruses replicate during person-to-person transmission (circulating VDPV: cVDPV) or persistent infections in immune deficient individuals (immunodeficiency-related VDPV: iVDPV) [6]. Nine nt substitutions are consistent with prolonged infection after receiving the tOPV dose. Attenuation of neurovirulence in OPV2 is conferred by nt 481 in the 5’ untranslated region (UTR) and the nts that encode amino acid 143 in VP1 [7]. Both nt 481 and amino acid 143 had reverted to wild type. It is important to stress that at no stage did the patient exhibit symptoms of paralytic poliomyelitis (acute flaccid paralysis: AFP), thus this situation would have been missed by classic AFP surveillance. Oral use of gamma globulin product did not yet clear this prolonged poliovirus infection.

## Measures to prevent onward transmission and follow-up

Upon notification of the poliovirus infection, the child was transferred to a contact isolation room requiring entrance with disposable gown and use of gloves and stools are monitored monthly. All visitors receive an explanation of the child's condition and instructions on hand hygiene.

Eight stool samples taken approximately once every month, including one from this August, have remained VDPV2-positive and the virus has continued to evolve. Important information can be derived from the temporal pattern of nt and inferred amino acid substitutions that persist or are transitory during early stages in the establishment of persistence. Such changes are highlighted in the Table in yellow and green, respectively for isolates from our SCID patient.

**Table:** Nucleotide and amino acid changes in viral protein 1 over time in immunodeficiency-related vaccine-derived poliovirus isolated from the stools of a severe combined immune deficiency patient, Israel, October 2015–August 2016

Sabin 2 sequence		Immunodeficiency-related vaccine-derived poliovirus type 2 isolate number Approximate number of days after last trivalent oral poliovirus vaccine							
		1	2	3	4	5	6	7	8
		119	172	200	247	292	334	382	409
**Position**	**Nt**	**Nt substitutions^a^**							
10	G	None	None	None	None	None	None	None	T
26	C	T	T	T	T	T	T	T	T
40	A	G	G	G	G	G	G	G	G
44	A	None	None	None	G	G	G	G	G
55	G	None	None	None	None	R	None	None	None
81	G	None	None	None	A	R	R	None	None
103	C	None	None	None	None	None	None	T	None
117	G	A	A	A	A	A	A	A	A
288		None	None	None	None	None	None	None	R
308	G	A	A	A	A	A	A	A	A
364	C	T	T	T	T	T	T	T	T
405	T	None	None	None	None	None	Y	None	None
**428^b^**	**T**	**C**	**C**	**C**	**C**	**C**	**C**	**C**	**C**
459	A	None	None	None	None	None	R	G	G
486	C	None	None	None	T	T	T	T	T
501	T	None	None	None	None	None	Y	None	None
516	C	T	T	T	T	T	T	T	T
540	C	None	None	None	None	Y	Y	None	None
600	A	None	None	None	None	None	W	None	None
660	A	None	None	None	None	None	None	R	None
769	A	G	G	G	G	G	G	G	G
849	T	A	A	A	A	A	A	A	A
**Total Nt changes**		9	9	9	12	14	17	14	14
**Position**	**AA** **(codon)**	**AA substitutions** **(codon^a^)**							
4	D (GAC)	None	None	None	None	None	None	None	Y (TAC)
9	A (GCC)	V (GTC)	V (GTC)	V (GTC)	V (GTC)	V (GTC)	V (GTC)	V (GTC)	V (GTC)
14	T (ACT)	A (GCT)	A (GCT)	A (GCT)	A (GCT)	A (GCT)	A (GCT)	A (GCT)	A (GCT)
15	K (AAA)	None	None	None	R (AGA)	R (AGA)	R (AGA)	R (AGA)	R (AGA)
19	V (GTT)	None	None	None	None	I/V (RTT)	None	None	None
35	P (CCA)	None	None	None	None	None	None	S (TCA)	None
103	R (AGA)	K (AAA)	K (AAA)	K (AAA)	K (AAA)	K (AAA)	K (AAA)	K (AAA)	K (AAA)
**143^b^**	I (ATT)	T (ACT)	T (ACT)	T (ACT)	T (ACT)	T (ACT)	T (ACT)	T (ACT)	T (ACT)
257	I (ATC)	V (GTC)	V (GTC)	V (GTC)	V (GTC)	V (GTC)	V (GTC)	V (GTC)	V (GTC)
**Total amino acid changes**		5	5	5	6	7	6	7	7

AA: amino acid; Nt: nucleotide.

Cells in green represent transitory nt or inferred amino acid substitutions while cells in yellow indicate substitutions that persist in all subsequent isolates. When a mutation is first detected in the latest isolate obtained, the cell is not shaded as it is remains to be seen whether this mutation will be found in further isolates.

^a^ R = A and G; Y = C and T; W = A and T.

^b^ Neurovirulence attenuation site.

The patient will continue to be monitored monthly until cessation of infection is documented [8] by two successive VDPV2 monthly samples. Prolonged infection either ceases spontaneously, in some cases after BMT, or becomes persistently established [9]. The patient can remain asymptomatic for years [9,10], develop poliomyelitis, or die from poliovirus or non-poliovirus related causes [9].

As iVDPVs continue to diverge, accumulating numerous amino acid substitutions in receptor binding epitopes and neutralising antigenic epitopes, the probability for transmission appears to decrease [9,11]. To date, there is only one documented case of transmission of iVDPV [12], but none for very highly diverged VDPVs [11]. This may be due in part to their need to adapt for persistence in a specific sub-region of the gut. The simultaneous presence of different lineages of highly diverged polioviruses in a PID patient without evidence of interaction (no recombination) [9,13] and from environmental surveillance samples containing polioviruses presumably excreted from a different unidentified single individual [5] suggests replication of the different lineages in separate locations and supports specialisation which may come at the expense of transmissibility. However, early in the process of iVDPVs’ adapting for persistence, the genome of the virus is most similar to the parent OPV strain and newly emerging cVDPVs and could presumably spread within the general population as cVDPVs can [9]. Moreover initial mutations tend to restore fitness to vaccine strains and reverse attenuation for neurovirulence [9,11,14] as in the current case we present.

## Epidemiological implications

During this eight-month interval, GAP III restrictions governing use of PV2 in non-essential laboratories came into force. GAP III provides clear instructions for containing PV2 and mitigating its transmission from laboratories [3]. However, no such global restrictions or general standard operating procedures exist for handling of persistent or prolonged infection of PIDs in a closed hospital setting where there may even be a higher risk of transmission through staff, family, or other close contacts to other PID patients or to the general population. The same four conditions that were of concern for transmission in poliovirus laboratories [15] occur in paediatric wards for immune deficient patients and raise concern for heightened risk of re-emergence of PV2 from this source during the critical transition time to a PV2-free world. Namely: (i) high concentrations of VDPV2, primarily in stools but possibly also respiratory samples are present; (ii) repeated exposure to high concentrations of poliovirus over long periods of time in a closed setting by attending medical, janitorial and laundry staff, equipment maintenance staff, family members especially those who stay overnight with their children, and specialised procedure medical teams; (iii) susceptibility of these primary contacts to infection and especially other naïve PID patients in the same ward who might be exposed through shared primary contacts and who lack an immune system capable of protection against infection and disease; and (iv) the general community protected from disease, but less so against infection.

## Conclusions

Re-emergence of VDPV2 from PIDs will be difficult to detect since infection of the immediate professional staff will be asymptomatic due to vaccination and most community infections are also likely to be asymptomatic when vaccine coverage is very high, such as in Israel [14,16]. Two models of the sustained transmission of WPV type 1 (WPV1) in Israel in 2013–14 in the population that had >95% coverage with three doses of inactivated poliovirus vaccine (IPV), predicted a delay of at least one year before any AFP cases might appear [17,18] and in fact AFP surveillance failed to document the sustained asymptomatic transmission of WPV1 throughout this period of sustained transmission [14,16].

It is critical in this transition period to identify and contain all PIDs infected with and excreting PV2. For the reasons above, we strongly recommend active paediatric PID stool surveillance at least of patients with a recent history of OPV vaccination even though a number of studies indicate that prolonged excretion among PIDs is rare [8,11,19]. The need to screen PIDs will decrease as the transition time from the tOPV to bOPV increases. There is also an urgent need for global instructions on how to care for these patients and how to monitor contacts.

## Ethics statement

The Ethical Review Board of the Sheba Medical Center, Tel Hashomer, approved this study (SMC-3412–16) and exempted it from a requirement to obtain informed consent.

## References

[1] Global Polio Eradication Initiative (GPEI). Global eradication of wild poliovirus type 2 declared. GPEI; 2015.

[2] World Health Organization (WHO). World Health Assembly resolution: poliomyelitis. Geneva: WHO; 2015. Sixty-Eighth World Health Assembly, May 26, 2015. Resolution no. WHA 68.3.

[3] World Health Organization (WHO). WHO Global Action Plan to minimize poliovirus facility-associated risk after type-specific eradication of wild polioviruses and sequential cessation of oral polio vaccine use (GAPIII). Geneva: WHO; 2015.

[4] Centers for Disease Control and Prevention (CDC). Laboratory surveillance for wild poliovirus and vaccine-derived poliovirus, 2000-2001. MMWR Morb Mortal Wkly Rep. 2002;51(17):369-71. 12018383

[5] Shulman LM Manor Y Sofer D Handsher R Swartz T Delpeyroux F Neurovirulent vaccine-derived polioviruses in sewage from highly immune populations. PLoS One. 2006;1(1):e69. 10.1371/journal.pone.0000069 17183700 PMC1762338

[6] Jorba J Campagnoli R De L Kew O. Calibration of multiple poliovirus molecular clocks covering an extended evolutionary range. J Virol. 2008;82(9):4429-40. 10.1128/JVI.02354-07 18287242 PMC2293050

[7] Sutter RW, Cochi SL, Melnick JL. Live Attenuated Poliovirus Vaccines. In: Plotkin S, Orenstein WA, editors. Vaccines, Third Edition. Philadelphia: W.B. Saunders Company; 1999. p. 364-408.

[8] Li L Ivanova O Driss N Tiongco-Recto M da Silva R Shahmahmoodi S Poliovirus excretion among persons with primary immune deficiency disorders: summary of a seven-country study series. J Infect Dis. 2014;210(Suppl 1):S368-72. 10.1093/infdis/jiu065 25316857

[9] Kew OM Sutter RW de Gourville EM Dowdle WR Pallansch MA. Vaccine-derived polioviruses and the endgame strategy for global polio eradication. Annu Rev Microbiol. 2005;59(1):587-635. 10.1146/annurev.micro.58.030603.123625 16153180

[10] Dunn G Klapsa D Wilton T Stone L Minor PD Martin J. Twenty-Eight Years of Poliovirus Replication in an Immunodeficient Individual: Impact on the Global Polio Eradication Initiative. PLoS Pathog. 2015;11(8):e1005114. 10.1371/journal.ppat.1005114 26313548 PMC4552295

[11] Burns CC Diop OM Sutter RW Kew OM. Vaccine-derived polioviruses. J Infect Dis. 2014;210(Suppl 1):S283-93. 10.1093/infdis/jiu295 25316847

[12] Alexander JP Ehresmann K Seward J Wax G Harriman K Fuller S Vaccine-Derived Poliovirus Investigations Group. Transmission of imported vaccine-derived poliovirus in an undervaccinated community in Minnesota. J Infect Dis. 2009;199(3):391-7. 10.1086/596052 19090774

[13] Yang CF Chen HY Jorba J Sun HC Yang SJ Lee HC Intratypic recombination among lineages of type 1 vaccine-derived poliovirus emerging during chronic infection of an immunodeficient patient. J Virol. 2005;79(20):12623-34. 10.1128/JVI.79.20.12623-12634.2005 16188964 PMC1235840

[14] Shulman LM Mendelson E Anis E Bassal R Gdalevich M Hindiyeh M Laboratory challenges in response to silent introduction and sustained transmission of wild poliovirus type 1 in Israel during 2013. J Infect Dis. 2014;210(Suppl 1):S304-14. 10.1093/infdis/jiu294 25316849

[15] Dowdle WR Gary HE Sanders R van Loon AM. Can post-eradication laboratory containment of wild polioviruses be achieved? Bull World Health Organ. 2002;80(4):311-6. 12075368 PMC2567771

[16] Kaliner E Kopel E Anis E Mendelson E Moran-Gilad J Shulman LM The Israeli public health response to wild poliovirus importation. Lancet Infect Dis. 2015;15(10):1236-42. 10.1016/S1473-3099(15)00064-X 26213249

[17] Kalkowska DA Duintjer Tebbens RJ Grotto I Shulman LM Anis E Wassilak SG Modeling options to manage type 1 wild poliovirus imported into Israel in 2013. J Infect Dis. 2015;211(11):1800-12. 10.1093/infdis/jiu674 25505296 PMC7887763

[18] Yaari R Kaliner E Grotto I Katriel G Moran-Gilad J Sofer D POG group. Modeling the spread of polio in an IPV-vaccinated population: lessons learned from the 2013 silent outbreak in southern Israel. BMC Med. 2016;14(1):95. 10.1186/s12916-016-0637-z 27334457 PMC4918056

[19] Guo J Bolivar-Wagers S Srinivas N Holubar M Maldonado Y. Immunodeficiency-related vaccine-derived poliovirus (iVDPV) cases: a systematic review and implications for polio eradication. Vaccine. 2015;33(10):1235-42. 10.1016/j.vaccine.2015.01.018 25600519 PMC5529169

